# Success of Process Innovations Through Active Works Council Participation

**DOI:** 10.3389/fpsyg.2022.795143

**Published:** 2022-04-08

**Authors:** Kai Breitling, Wolfgang Scholl

**Affiliations:** ^1^Department of Social Security Administration, Federal University of Applied Administrative Sciences, Berlin, Germany; ^2^Department of Psychology, Faculty of Life Sciences, Institute for Psychology, Humboldt University of Berlin, Berlin, Germany

**Keywords:** innovation success, process innovations, co-determination, employee participation, works council, coordination capability, knowledge growth, path analysis

## Abstract

Successful innovations are deemed to be necessary requisites for enterprise success. On the other hand, works council participation (“co-determination” in Germany) and employee participation are judged differently as either fostering employee and enterprise benefits or only the former or even none. Both forms of participation have found diverging theoretical and empirical argumentations regarding innovations. Here, we argue and show empirically that both forms of participation deliver positive contributions to innovation success, economically and employee-related, substantiated with qualitative reports from 36 process innovation cases and quantitative data from 44 cases. Qualitative case analyses reveal different profiles of works council participation depending on the innovation type. Independent of the innovation types, more successful innovations are marked by more intensive participation. Quantitative examinations of a causal model with path analysis specify how this is achieved: works council and employee participation further the growth of appropriate knowledge and the former also raises the coordination capability; both are essential preconditions for innovation success. A direct impact of works councils on innovation success complements the indirect effects. The slightly modified path analysis explains 53% of the innovation success variance.

## Introduction

Innovation is considered to be a key factor of organizations’ success and prosperity (Banbury and Mitchell, 1995; Brockhoff, 1999; Artz et al., 2010). Thus, the question of what drives innovation is widely discussed. Research has focused on economic parameters as well as on social factors to predict innovation success. Among the wide range of social factors, participation has been identified as having a vital influence on the innovation process (West and Farr, 1990; Boonstra and Vink, 1996; Michie and Sheehan, 1999; Spreitzer et al., 1999; De Dreu and West, 2001; Tonnessen, 2005). Participation is referred to as the involvement of employees in decision-making and can be found in various forms: while *direct* participation is defined as the “immediate, personal involvement of organization members in decision making” (Dachler and Wilpert, 1978, p. 12), *indirect* participation is performed on behalf of the employees by an elected committee of co-workers. In Germany, *indirect* participation is legally prescribed as so-called *co-determination*: employees are represented by a works council on the factory level, as well as by employee representatives on the board of larger corporations. We will refer to direct participation as *employee participation* and to indirect participation as *works council participation* or *co-determination.*

The positive contribution of employee participation and co-determination to employee interests is hardly contested (Scholl and Kirsch, 1986; Wagner, 1994). In fact, Weber et al. (2020) confirmed an increase in job satisfaction, work motivation, employees’ organizational commitment, and the perceived supportive climate in the organization. By contrast, the impact of works councils on innovations concerning business practices is not as clear. One reason for the heterogeneous evidence is the diversity of national co-determination systems for which most countries provide their own legal framework which defines how much influence works councils can have on management decisions. In Europe, as a result of different cultural, political, and historical backgrounds, co-determination rights and their application vary substantially between countries (Knudsen, 1995; European Commission [EC], 2008). To overcome these national differences, the European Union has been taking measures to harmonize legislation and achieve a deeper integration of its member states. One major step in this process was the EU’s 1994 European Works Council directive (European Commission [EC], 2011), but integration efforts are still ongoing. For future modifications of the European legislation, it is essential to generate more scientific data about the industrial relations systems of the member states to facilitate a more accurate assessment of their advantages and disadvantages. This study aims to provide deeper insights into the actions and consequences of German works councils in process innovations, which may provide further knowledge about the mechanisms of works council participation and give new directions for European legislation regarding works councils. The study comprises 45 innovation cases based on interviews with management and works council members as well as standardized questionnaires filled in by the same respondents.

## Works Councils – Promoters or Inhibitors of Innovations?

When we speak of innovations in this article, we refer to process and organizational innovations. Process innovations are “new or significantly improved production or delivery methods,” including “new equipment, software, and specific techniques or procedures” (Organisation for Economic Co-Operation and Development [OECD], 2005, p. 55), whereas organizational innovations are “new organizational methods in business practices, workplace, organizational, or external relations” (Organisation for Economic Co-Operation and Development [OECD], 2005, p. 55). We focus on these types of innovation because they usually bring a significant change of working conditions and other employee-related outcomes. According to the German Works Constitution Act, management decisions that directly affect the workplace are strongly subject to the co-determination of the works council. Thus, organizational and process innovations are more relevant to our research question than product or service innovations.

The effect of co-determination on innovations has been a source of scientific controversy. There exist two theories about the influence of works councils on innovations that make contrary predictions: new institutional economics (NIE) on the one hand and participation theory on the other (for detailed reviews of this discussion see Dilger, 2002; Hucker, 2008; Renaud, 2008). Supporters of the NIE approach contend that works councils inhibit innovations by virtue of their assumed primary goal of maximizing wages at a minimum amount of work. According to NIE, the consequences of co-determination for the company are threefold: increased transaction costs due to more extensive wage bargaining, reduced profits, and finally, weakened employers’ property rights. These factors are expected to have an overall negative effect on firm innovation because capital owners will stop spending money on innovations if chances of a poor return on investment are high (Jirjahn, 2011).

In contrast, advocates of the participation theory argue that works councils promote innovations instead of inhibiting them. The positive influence of representative participation comes from the works council’s role as a collective voice of the workforce (Freeman and Medoff, 1984; Freeman and Lazear, 1995). Without a works council, employees have to talk to management or their superiors, personally, if they want to express dissatisfaction or make suggestions for improvements, which exposes them to the possibility of being sanctioned. In contrast, works councils cannot be sanctioned by the employer and are covered by very strong lay-off protections; for them, there is no reason to hold back demands or to bring them forward in an overly cautious way. According to the participation theory, works councils - by speaking on behalf of the employees - enhance the exchange of information over hierarchy levels, especially from the bottom upward. Knowledge at the operational/shop floor level penetrates the organization more easily and is more likely to be considered in management decisions. As a result, innovativeness is expected to increase because organizational knowledge is better exploited, problems are detected earlier, and the quality of management decisions eventually improves.

It is difficult to decide on mere theoretical grounds which approach is more appropriate to predict the factual influence of co-determination on innovations. NIE has been criticized for its one-sided and monolithic view of employers’ and employees’ motives (Streeck, 2008). In fact, findings from motivational and organizational psychology about workplace behavior have been widely ignored by the NIE approach. More specifically, employees as well as their representatives have an interest in the long-term success of their firm because their workplaces depend on it. Yet as a third possibility, co-determination might have simultaneous negative and positive effects on innovation, which would make it difficult to predict the net result (Bertelsmann-Stiftung and Hans-Böckler-Stiftung, 1998).

To find empirical support, a substantial number of studies on the relationship between works council participation and innovativeness have been conducted. Despite the methodological diversity of these studies, they allow some insights. In the first group of studies, the authors examine the impact of the mere presence of works councils on innovations. The findings show either no correlation (Blume and Gerstlberger, 2007; Hempell and Zwick, 2008) or a positive correlation (Hübler and Jirjahn, 2002; Zwick, 2004) between the existence of a works council and workplace innovations. These heterogeneous results still reveal a common outcome of these studies: the lack of a negative effect of works councils (see also Wilpert, 1998, p. 63; Addison et al., 1999, p. 241). This can be considered the first hint toward our research question, but definitely calls for a deeper look into the underlying processes of co-determination.

Other authors take the diversity of works councils into account. There are two dimensions commonly referred to specify this diversity: (a) the negotiation strategy of works councils, which can be either conflict-oriented or consensual, and (b) the works councils’ strength or proactivity, which can range from passive-reactive to proactive and self-initiated. Various works council typologies are based on one or both of these dimensions (Kotthoff, 1981, 1994; Müller-Jentsch and Seitz, 1998). Research findings confirm a close relationship between works councils’ strength and innovation output: Organizations with a strong and proactive works council have a clear lead in innovation over those with passive and reactive works councils (Nienhüser, 2005; Ziegler et al., 2010). At the same time, the conflict style of works councils seems to play no major role in innovation, since no or only weak correlations have been found (Frick, 2002; Nienhüser, 2005).

In most of the cited studies, innovativeness is defined as the number of new methods and practices (i.e., innovations) implemented in a certain time interval. This approach ignores the fact that many innovations fail because they do not achieve the expected goals of better performance and/or working conditions (Klein and Knight, 2005). In line with other authors, we took these outcomes into account to assess innovation success more adequately. However, there is currently no research that explicitly investigates the relation between co-determination and innovation outcome indicators. We tackled this question empirically in our study based on the positive interest of employees and their works councils into the firm’s long-term success as well as on the above-cited literature. Our research hypotheses try to depict more closely the main processes which relate works council participation to innovation success. The first hypothesis in the research proposal read:

(H1) The stronger the works council participation during the innovation process, the stronger is its positive effect on the innovation regarding its efficiency, quality of working life, and overall effectiveness.

### How Are Innovations Affected by the Co-determination of Works Councils?

Participation is sometimes referred to as a black box, because not much data exists as to *why* it has an impact (vgl. Junkes and Sadowski, 1999, p. 84; Torka et al., 2008). Many studies show correlational evidence for the link between innovation and co-determination but do not provide information about the mediating processes. The explanations of why works councils have an effect are mainly based on theoretical assumptions rather than on empirical data. Participation theory argues that the benefit of works councils on innovations is mediated by an enhanced exchange of knowledge within the organization (see above). In fact, a study by Klippert et al. (2009) shows that a high degree of cooperation between the works council and management correlates with more effective knowledge management structures in the organization. At the same time, these knowledge management structures are closely related to successful product and process innovations (Blume and Gerstlberger, 2007). Similarly, Scholl (2004) reports that the more knowledge is gained during IT-related innovation processes through participation, the more successful are these innovations.

Apart from knowledge exchange, another variable is to be considered for mediating the influence of co-determination on innovation: the capability of the organization to coordinate the ideas, interests, and actions of the persons involved in the innovation process. Coordination capability is considered essential for dealing with complex organizational processes like innovations. It comprises two dimensions: the *ability of decision-making* and the *ability of implementation* (Scholl and Kirsch, 1986; Scholl, 2004). While it is often argued by NIE proponents that co-determination weakens the coordination capability, empirical data show the contrary: strong co-determination *facilitates* and improves the coordination capability (Bartölke et al., 1982; Scholl and Kirsch, 1986). When dealing with a strong works council, management is less inclined to push forward their own ideas and instead will pay more attention to employees’ concerns and the complexity of the whole innovation process (Scholl and Kirsch, 1986). If this complexity is ignored, subsequent problems, as well as more resistance, are more likely, which impairs the coordination capability more severely than when involving the works council at an early stage of the innovation process. Several studies confirm with path analyses that coordination capability is an essential success factor for innovation (Scholl, 2004, 2019; So, 2010). Therefore, we hypothesize: The promotional influence of co-determination on innovation success is – at least in part – mediated by knowledge growth and coordination capability. In detail:

(H2) The stronger the works councils’ participation, the stronger is its positive effect on knowledge growth.

(H3) The stronger the works councils’ participation, the stronger is its positive effect on the coordination capability during the innovation process.

(H4) The higher the knowledge growth, the more positive will be the effect on the innovation’s success with regard to efficiency, the quality of working life, and overall effectiveness.

(H5) The higher the coordination capability, the more positive will be its effect on the innovation’s success with regard to efficiency, the quality of working life, and overall effectiveness.

### Co-determination as a Remote or Proximate Factor?

Co-determination, as well as other variables in social science, can be operationalized either on a remote or a proximate level. A remote factor is considered a context variable, assuming that it does not vary much over time and is hardly influenced by other factors (Schneider and Wagemann, 2006). In participation research, this approach can be found in studies on how the presence or absence of a works council affects organizations. Typologies of works councils, too, take a remote perspective. The categories set up to characterize works councils imply that they always act in a similar way without being affected by situational factors or the persons involved. In contrast, a proximate factor varies over time and is influenced by the situational setting and involved persons. Thereby, it is temporarily and spatially linked much more closely to the outcome (Schneider and Wagemann, 2006). A proximate view on co-determination is attained by examining the concrete behavior of works councils in specific situations or during specific events. Schneider and Wagemann (2006) as well as Kitschelt (2003) recommend distinguishing between remote and proximate aspects to analyze causal *structures* (remote) on the one hand and causal *mechanisms* (proximate) on the other. As for co-determination, studies confirm the relevance of this distinction (although the authors do not explicitly refer to the remote-proximate concept). For example, Osterloh (1993) found that the amount of information provided by management could be predicted much more precisely based on how the works council was involved in joint decision-making (proximate factor) than by the co-determination law applicable (remote factor). Similar results were reported by Horsmann and Pundt (2008). Thus, we assume that the remote aspect of co-determination, i.e., the general characteristics of a works council is more predictive for its specific participation in the innovation process than for the innovation success itself. So, we hypothesize:

(H6) The stronger the overall influence of the works council within the company, the stronger is its effect on the works council participation during the innovation process.

**FIGURE 1:**
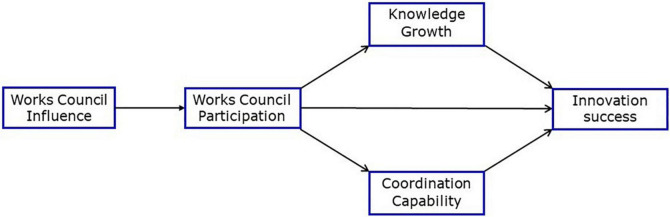
Path model of works council participation in innovation processes.

These six hypotheses build a small causal model which shall be tested with a path analysis (see Figure 1). For instance, the combination of H6 with the hypotheses H1 to H5 means that works council participation mediates the overall influence of the works council *via* three paths onto innovation success.

### Works Council Participation vs. Employee Participation

German law provides the right for employees to found and establish a works council in their company if the total number of employees is more than five. Yet, if there are no candidates that are willing to run for this honorary office, then there will be no works council. In fact, only about 10% of German companies are co-determined by a works council; this rate ranges from 6% in small enterprises up to 90% in large companies with over 500 employees (Ellguth and Kohaut, 2010). In contrast, in all organizations, there is some degree of employee participation because most management decisions require the involvement of subordinate employees at a certain point to be executed. So, in co-determined organizations, we always find employee and works council participation coexisting. But how do they interact in the innovation process? Before answering this question, it has to be noted that, in the participation literature from scientists and practitioners, the term ‘employee participation’ has become a special term for extra-role participation or more-than-usual participation. In our qualitative and quantitative measurements, we see and register both normal role participation (e.g., general information to the workforce) and extra-role employee participation (e.g., ‘Employees contributed, continually, new ideas and suggestions’).

For employee participation, several authors show a positive effect on innovations (West and Farr, 1990; Wengel and Wallmeier, 1999; West, 2002; Scholl, 2004; Schepers and van den Berg, 2007). Similar to the participation of works councils, the involvement of employees is supposed to provide easier access to relevant knowledge at the operational level (Lawler, 1992; Scully et al., 1995; Wilpert, 1998) and to reduce resistance to change (Coch and French, 1948; Vahs, 2007, p. 343). However, no data is available about how the *combination* of employee and works council participation affects innovation. Research in the field of industrial relations provides evidence mostly about how they affect each other. In the 1970s and 1980s, unions and works councils suspected that employee participation under management control is a rival to co-determination (Leminsky, 1998, p. 58). Yet recent findings do not confirm this position and instead endorse the theory of mutual enhancement (Dörre, 1996; Fröhlich and Pekruhl, 1996, chap. 6; Kotthoff, 1998; Helfen, 2005; Tietel, 2006; Pohler and Luchak, 2014). These findings still make it difficult to deduce implications for innovations. They allow only for the minimal assumption that the coexistence of works council and employee participation does not impair the innovation success. Therefore, we do not propose any specific hypothesis. Instead, we statistically explore how employee participation relates to works council participation as well as to innovation outcomes and the mediating variables. Considering earlier studies, we expect:

(E1) There is a positive effect of employee participation on the innovation process which has to be specified.

The combination of these hypotheses and expectations shall be tested with structural equation models, respectively, with path analyses (see below).

### What Is the Contribution of Works Councils in the Innovation Process?

To draw a clearer picture of the mechanisms of co-determination, it is not sufficient to investigate the data using a quantitative questionnaire. Standardized measures of co-determination offer only a limited view because they are focused on very few dimensions, e.g., extent of participation or conflict style of works councils. These dimensions are too abstract to describe what works councils actually do in the innovation process, especially if one considers the multitude of issues attached to innovation projects on the one hand and the available options for dealing with them on the other. For a deeper understanding, it is necessary to add a qualitative view on co-determination by examining the content of works council activities.

Authors using a qualitative approach report a variety of issues works councils are concerned with in the innovation process. In the 1970s, unions in Germany began to discuss innovation more deeply (e.g., Hinz, 1978) as a possible alternative to rationalization. The German Metalworkers’ Union (IG Metall) founded the local Innovation Consulting Centers which offered information and practical help for works councils about all aspects of innovation, including societal and ecological aspects (Klotz, 1987). It was explicitly intended as an alternative strategy to shape the selection and content of innovations in a socially responsible way. This includes all the topics that come along with innovations, their primary goal, technical and organizational feasibility, financial risks, and their consequences for employee-related concerns. Despite this variety, works councils focus primarily on those topics which are considered to be most closely linked to their core responsibility: the well-being of the employees (Bartölke et al., 1991; Stracke and Nerdinger, 2010), e.g., job security, health and safety at work, company social services, re-training, etc. (see the respective paragraphs in the Works Constitution Act). Thus, they try to cope with the expected consequences of the innovation for the staff members rather than shaping the innovation process properly. Nevertheless, works councils also get involved in the innovation scope itself. Process innovations in particular more frequently deal with technical and organizational changes of the company than directly with employee affairs, but often they have consequences for the latter, too. Depending on their expertise and demand for participation, works councils may get involved in any issue of innovation. Their involvement may range from critically observing the innovation process, intervening from time to time, making suggestions for improvement or even initiating and elaborating their own innovation concepts (Rundnagel, 2004; Ziegler et al., 2010; Schwarz-Kocher et al., 2011). Another, but rather indirect way for works councils to influence the innovation, is to enhance the context parameters (Kern and Schumann, 1984; Stracke and Nerdinger, 2010), for example by helping to eliminate organizational barriers, increasing employees’ motivation and their capacity to continually improve the company, or arranging incentives for change. Finally, works councils can also oppose or completely reject an innovation. This is only an option of last resort, which can be necessary if employee interests are seriously threatened through the innovation (by layoffs, poor working conditions, etc.). It is hardly possible to predict how widely-used these different forms of works council participation in innovation processes really are and which consequences they have. Therefore, we want to qualitatively explore innovation processes with the following research questions (RQ):

(RQ1) What are the aspects of the innovation that are relevant for works council participation, and to what kind of actions do they prompt works councils within the innovation process? Aspects can be any content of the innovation, any facet of the process, or any action of management related to the innovation at stake. Like managers, works councilors have limited resources of time, knowledge, and external support, so they have to prioritize their possible actions.

(RQ2) Are there specific patterns of works council action for different types of process innovations? Process innovations can be very different, e.g., comparing structural changes with human resource improvements. So, we look for possible types of innovation which comprise similar goals and action fields but differ a lot from other types.

(RQ3) Are there specific patterns of works council action for successful innovations in contrast to less successful ones? Since H1 assumes a positive effect of works council participation on innovation outcomes, it can be expected that co-determination varies not only quantitatively but also qualitatively with innovation success.

## Materials and Methods

The following study is based on a research project (conducted by the authors) with the title “Innovativität durch Mitbestimmung, untersucht an der Beteiligung von Betriebsräten an Prozessinnovationen” (Innovativeness through co-determination, investigated with regard to the participation of works councils in process innovations). It was financed by the Hans–Böckler–Stiftung (the foundation of the German Trade Union Confederation) after a competitive request for proposals. The foundation’s advisory committee was informed three times about the progress of the study while the independence of the researchers was not challenged. The final report was published by Scholl et al. (2013).

### Sample and Data Collection

To examine the effect of works council participation on innovation success, we combined quantitative and qualitative methods. We draw upon a case-based sample of 45 organizational and process innovations that were introduced no more than 5 years prior to our survey. Due to the limitations of field research, it was not possible to make up a randomized sample, so we had to select innovation projects according to certain criteria. The sampling process comprised two steps. In the first, we identified suitable companies. To avoid effects caused by different legal frameworks of co-determination, we concentrated on joint-stock companies and limited companies with a workforce of more than 500 employees. This made sure that in the supervisory boards, employee representatives had a mandatory share of one-third or – in the case of the workforce exceeding 2,000 employees – of half of the seats. In addition, we targeted a wide spectrum of economic sectors, so we included in our search various industrial and service branches with the presence of various unions. After a company had given its approval to participate in our research, we had to identify the innovations to be examined as a second sampling step. Our selection criteria aimed at composing a sample of innovations that featured considerable variance with respect to the (a) type/content of innovation, (b) extent of works council participation during the innovation process, and (c) innovation success (for b and c we applied rule of thumb estimates which were later assessed more precisely by our questionnaires and interviews).

The sample size of 45 cases should allow statistical analyses and still be small enough to allow in-depth analyses of every single innovation. To get a comprehensive and balanced view, we collected data both from the works council and from management about each selected innovation. For the qualitative section of our study, we conducted an interview with at least one participant from each side. Following the interviews, the interviewees were asked to fill in a standardized questionnaire for the comparative quantitative analyses. Both the interviews and the questionnaires aimed at tracing the evolution of each innovation process, thereby focusing on the participation of the works council, the employees, and their interaction with management. Interview data from both parties (works council and management) are available in 36 cases, questionnaire data in 30 cases. In 14 additional cases, the questionnaire was completed by one side only, in seven cases by a management representative and in seven cases by a works council member. For one case, only interview data were provided, which gives quantitative data on 44 cases.

### Qualitative Procedure and Analysis

The interviews aimed at getting a basic understanding of the works councils’ and the employees’ participation in process innovations. Well-informed members of management and of the works council were contacted for so-called expert interviews (Meuser and Nagel, 2009). A guideline was developed and used in the interviews with questions: (1) about the interviewed person, their function in the firm, and their professional role conception, (2) about the important aspects of the innovation project, (3) about the kind of participation of the works council and the employees in the innovation process, and (4) about the final assessment of the innovation, including its success. Based on a written data privacy contract, the interviews were audio-taped with the consent of the interviewees, except in 4 cases that could only be analyzed from interviewer notes. Most interviews lasted approximately 90 min.

As intended, the obtained innovations differed a lot in their characteristics. Therefore they were categorized into four groups according to their scope to examine whether the innovation type influenced the activities of the works council: (a) 10 innovations dealt with changes in the structure of the firm, e.g., rearranging business units and divisions, (b) 9 innovations dealt with changes in procedures and workflows, e.g., optimizing time and costs of processes, (c) 20 innovations concerned human resources, e.g., leadership methods, pension plans, compensation schemes, and (d) 6 innovations were about introducing new software, e.g., for accounting. Due to the complexity of innovation processes, it was not surprising that in some cases we found characteristics of more than one innovation type. However, for an unambiguous categorization, we referred to the initial trigger and/or primary goal of the innovation. For example, if new software was to be introduced and as a consequence, this required organizational adjustments or staff training, we still considered it a *software innovation* and not, e.g., a human resources innovation, because it was the new software that shaped and coined the whole innovation process.

The analysis of the interviews was based on *qualitative content analysis* (Mayring, 2014). This is a rather formal methodological approach, as it requires a category system that allows for the classification of contents as well as for a direct comparison of cases. Before the analysis is carried out, the original interview transcripts are paraphrased and summarized. Therefore, it is the method of choice for relatively large amounts of text as was the case in our study. We applied a specific technique of qualitative content analysis called *content structuring* (Mayring, 2014, p. 104).

In the first step, we filtered out the passages that contained statements about and descriptions of the works council activities during the innovation process from each interview transcript. We focused on statements that were closely linked to our three research questions, i.e., statements on how much the works councils contributed to the innovation proper, which employee interests they addressed, whether they enhanced the context parameters of the innovation, and whether they opposed the innovation. In the next step, these passages were summarized “to reduce the material in such a way that the essential contents remain” (Mayring, 2014, p. 64). In the third step, the summarized passages of all interviews per case were condensed to a coherent and consistent case description. Interestingly, none of the cases involved major conflicting or contradictory statements of the interviewees. However, what we were faced with rather often was the fact that interviewees mentioned certain details that others did not. These details were added up neatly and without inconsistencies in the final case descriptions. When different points of view were apparent on occasion, they applied primarily to subjective aspects that were not significant for the analysis. This could be observed, for example, in case HR-01 (introduction of employee performance reviews, see Appendix): whereas the interviewees’ descriptions of the works council’s activities were very similar, their personal opinion on the same topic differed rather clearly. While the works council representative expressed her satisfaction, the management interviewee was rather critical, stating that due to the works council the innovation had become a “tortuous and exhausting process.”

The category system for the analysis of the works council participation was developed by deduction from participation research literature and by induction via newly gained insights from the case descriptions (for the complete category system see Table 1). We defined four broader dimensions to cover works council activities comprehensively. These dimensions were further subdivided so that it was possible to classify the works council participation more precisely and specifically within a dimension:

(1) Activities affecting the content and design of the innovation (Bartölke et al., 1991; Kern and Schumann, 1998; Rundnagel, 2004; Horsmann and Pundt, 2008; Ziegler et al., 2010), subdivided into six values according to the intensity of these activities (ranging from minimal contribution up to initiating ideas).
(2) Activities concerning employee-related issues (Stracke and Nerdinger, 2010; Schwarz-Kocher et al., 2011), subdivided into four values according to the scope of these activities.
(3) Activities affecting the general conditions under which the innovation project and/or its implementation were carried out, with the aim to provide a smooth course of action within the organization (Kern and Schumann, 1984; Stracke and Nerdinger, 2010; no further subdivisions within this dimension).
(4) Activities of resistance against the innovation (Kley, 2018), subdivided into two values according to the aim of resistance.

**TABLE 1:** Category system for the analysis of the works council (WC) participation during innovation.

Dimension of WC participation	Subdivision	Anchor sample
Activities affecting the content and design of the innovation	WC initiates the innovation	• WC comes up with own idea and turns it into an innovation project. • Innovation is shaped profoundly by WC’s impulses.
	WC participates continually over the course of the innovation	• WC is an equal member of the innovation’s steering committee and has substantial influence on its decisions.
	WC contributes to specific topics of the innovation	• WC identifies yet unregarded aspects of the innovation and comes up with its own suggestions or solutions.
	WC acts as devil’s advocate of the innovation	• WC warns about possible problems, questions decisions, and re-checks results.
	WC as watchful observer of the innovation	• WC is well informed about the innovation process at any time and closely monitors its progress.
	WC has a passive role in the innovation	• WC does not contribute any ideas to the innovation. • WC is restricted to its legally prescribed functions (e.g., formal approval of certain management decisions).
Activities concerning employee-related issues	WC defends employees’ interests	• WC averts disadvantages for the employees caused by the innovation (e.g., overtime and layoffs).
	WC advocates for more qualification of employees	• WC initiates on-the-job-training for a new software.
	WC communicates with employees about the innovation	• WC openly discusses with the employees about the consequences of the innovation or alternative solutions.
	WC enhances employees’ acceptance of the innovation	• WC highlights the necessity and benefits of the innovation • WC disproves employees’ concerns about the innovation.
Activities affecting the general conditions under which the innovation project is carried out with the aim to provide a smooth course of action within the organization (*no further subdivision*).		• WC improves the structure or processes of the innovation project, e.g., by constantly urging management to get ahead with the project.
Activities of resistance against the innovation	Resistance because of the content of the innovation	• WC openly tries to impede the innovation out of anticipated disadvantages for employees.
	Resistance as a negotiation tactic concerning other issues	• WC announces to reject the innovation in order to achieve management’s concession in a different field.

Additionally, for each subdivision, one or two typical “anchor samples” (Mayring, 2014, p. 95) were extracted from the case descriptions in order “to illustrate the character of those categories” (Mayring, 2014, p. 95; for details see Table 1).

### Quantitative Measurement and Analysis

For the questionnaire, we took advantage of existing questions and items from former research on participation and/or innovation (e.g., Wilpert and Rayley, 1983; Scholl and Kirsch, 1986; Scholl, 1996, 2014; Schimansky, 2006; So, 2010; Bentz, 2011). The questions for works council and employee participation developed by other authors had to be adapted to the context of our study. As an example, to assess the extent of works council participation, we used the Wilpert and Rayley (1983) items as a template. Yet Wilpert and Rayley provided only one response option, i.e., out of several items that describe certain degrees of participation the survey participant selects the one he or she deems most applicable. In that case, we preferred different degrees of participation to be assessed independently to gain richer data.

The questionnaire was pretested with 20 works councilors and slightly modified for the final version. In the following, the items for each construct are given, together with their reliability. While the items demonstrate content validity, their construct validity was already shown in the above-cited former studies and can be judged from the theoretically expected correlations, which should be significant (they are significant, cf. Table 2 in the “Results” section).

**TABLE 2:** Works council participation in different types of innovations.

Innovation type	Focus of works council activity
Human resources innovations	Content contributions ++ Threatened employee interests 0
Software innovations	Content contributions 0 Threatened employee interests ++ Context +
Structural changes	Content contributions 0 Threatened employee interests + Context +/Support +
Work flow changes	Content contributions + Threatened employee interests + Qualification 0

*++ strong focus, + medium focus, 0 not in focus.*

The extent of *works council participation in the innovation process* (proximate variable) was measured with the following items, tapping the full range of participation possibilities:

“How did the works council participate in the shaping of the innovation?

• The works council had to enforce his participation by legal means;
• The works council was only informed about decisions already taken;
• The works council was already informed in preparation of important upcoming decisions;
• The works council expressed its opinion in preparation of important decisions;
• Important decisions were altered because of ideas and suggestions of the works council;
• The ideas and suggestions of the works council were significantly incorporated into important decisions;
• Important decisions were partly based on own ideas and suggestions of the works council;
• Important decisions were initiated by the works council.”

The items were scaled from 0 = not at all true, via 3 = partly true, and up to 6 = fully true. The reliability (Cronbach’s alpha) is α = 0.82, after excluding the first item.

In a similar way, employee participation was measured with these items:

“To what extent did the relevant employees participate in the innovation process?

• The employees were only informed just before the realization of the project;
• The employees were only informed about decisions already taken;
• The employees were already informed in preparation of important upcoming decisions;
• The employees expressed their opinions in preparation of important decisions;
• Important decisions were altered because of ideas and suggestions of employees;
• Employees contributed continually new ideas and suggestions;
• The ideas and suggestions of the employees were incorporated into important decisions;
• The employees initiated interesting project developments.”

The scales were the same as above. Cronbach’s alpha is α = 0.88.

*Knowledge growth* was measured with the following introduction: “During the innovation process, to which extent … were new things probed and new experiences garnered?”, “… were the actual problems identified?”, “…was the complexity of the task understood in depth?”, “… were new insights gained?”, “… were ideas and experiences of others adopted?”, “… were errors detected and processes improved?” The items were scaled from 0 = not at all, via 3 = partly, and up to 6 = very much. Cronbach’s alpha is α = 0.89.

*Coordination capability* was measured with the general introduction: “What was your impression: how serious were the following problems during the innovation process?” “The innovation process stagnated and threatened to sand up,” “Discussions went endlessly round in circles,” “Urging decisions were taken with delay,” “Decisions taken were not or incorrectly implemented,” “Decisions taken were arbitrarily altered during the implementation,” “Decisions were implemented nominally without meaningful adaptation to the situation.” The items were scaled from 0 = not at all, via 3 = partly, and up to 6 = very much. Cronbach’s alpha is α = 0.86.

*Innovation success* was measured with items for economic outcomes, on the one hand, and benefits for the employees on the other. By measuring these two aspects, possible win-win solutions could be identified. The introduction for economic success read: “How do you rate the economic success of the innovation regarding … the adherence to the timeline of the project?”, “… the adherence to the budget frame?”, “… cost reductions?”, “… the expected practical benefit?”, “… the achieved problem solution?” The introduction for the employee-related benefits read: “How do you rate the consequences of the innovation for the employees regarding … the work place quality?”, “… the work climate?”, “… the wages?”, “… the opportunities for professional development?”, “… the employees’ autonomy at work?” The items were scaled from −3 = total failure down to −1 on the left side, 0 = partly, to +1 on the right, and up to +3 = total success. Cronbach’s alpha is α = 0.80.

The *overall influence of the works council* within the company (distal variable) was measured as a difference from the estimated influence of the top management, due to the fact that a leading works councilor could be also a member of the supervisory board controlling the top management. The relevant question asked for a general assessment of the time before the introduction of the innovation: “How strong or weak was the influence of the following groups on decisions in the company? Top Management/Middle Management/Works Council/Union,” to be estimated for each group from 0 = very weak up to 6 = very strong. Only the difference between top management and works council was used for the overall influence of the latter in the reversed form: the lower the difference, the higher the reversed value; no difference between management and works council influence would be 0, in reversed form 6. No reliability could be calculated from the single ratings.

As could be expected, the data varied not only between cases, but also between the assessments of the respondents of the same case: a works council member, a responsible manager, and/or a project leader. Differences in assessments might be partly due to different observations, experiences, and reports of different parts of the innovation and partly due to biases based on positional and personal interests. Thus, the correlations between the estimates from management and from the works council show medium to near-zero values: overall influence of the works council: *r* = *0.06*, works council participation: *r* = *0.40*, employee participation: *r* = *0.03*, coordination capability: *r* = *0.31*, knowledge growth: *r* = *0.06*; and innovation success: *r* = *0.26*. If different respondents get their impressions from different aspects of a longer innovation process, then averaging the responses is the best way to reach a reasonable picture of these co-determined innovations. Thus, the different impressions were averaged and complemented each other, which leads to a more realistic conclusion about the cases in question. So, first, the answers from a manager and a project leader (if both were available) were averaged, and secondly, the answers from management and the works council member were averaged. It can be expected that the resulting variables are more valid than the impressions of either side. The construct validity can be checked through the significance of the correlations between the averaged variables. The below reported empirical analyses confirmed this expectation (the same was already shown in Scholl and Kirsch, 1986, p. 360). Separate analyses for both groups are not useful because the kind and amount of biases cannot be assessed and compensated.

For the quantitative analysis of the hypotheses via path analysis, there were 6 unknown parameters (H1 – H6) to be estimated and one or two additional parameters if employee participation shall be included (E1). According to Bagozzi and Yi (2012), 5–10 cases per unknown parameter are necessary for a sufficient estimation, i.e., 35–40 cases are at least necessary for the intended path analysis. The 30 cases where questionnaires from both sides were available fall short of this minimum. Therefore, the 7 solitary questionnaires from management and the 7 from the works council were also used; the lack of the other side’s questionnaire was compensated for by using 10 multiple imputations with PASW Statistics. A comparison between the relevant correlation coefficients of the 30 cases from both sides and of the imputed 44 cases data set showed no significant differences. Moreover, the imputed data set is somewhat more conservative because most correlations are a bit lower than those from the 30 cases. With the 44 cases, we could use the maximum of available information for computing a correlation matrix out of the measured constructs as a sufficient basis for the intended path analysis.

Because of the fully specified theoretical model, significance tests will be one-sided: with *N* = 44, *t*-values > 1.30 are significant at *p* < 0.10 (^†^); *t* > 1.68: *p* < 0.05 (*); *t* > 2.42: *p* < 0.01 (**); *t* > 3.30: *p* < 0.001 (***). Only the expected but not precisely specified causal location of employee participation will be tested two-sided if it can be integrated at all: *t* > 1.68: *p* < 0.10 (^†^); *t* > 2.02: *p* < 0.05 (*); *t* > 2.70: *p* < 0.01 (**).

## Results

### Qualitative Analysis

As already outlined, the innovation cases were partitioned into four types: structural changes (SC), workflow changes (WF), human resources matters (HR), and new software introductions (IT). The actions of the works councils were analyzed with the category system presented in the methods section. A detailed presentation of all works council contributions in the 36 cases is not possible within the limits of this article. Therefore, a listing of all 36 innovations is given in the Appendix where the innovation types, the contributions of the works council, complemented with relevant employee activities (from Shajek, 2013), and the innovation success are shortly indexed and described. Here, we will only discuss the main results and insights which can be drawn from the general overview. As expected, the works councils’ foci varied substantially with the innovation type and innovation success.

#### Human Resources Innovations

The most intensive *content contributions* could be found in human resources innovations (indexed HR-01 to HR15, see Appendix), which is also the field with the most extensive co-determination rights and with the bulk of the usual works council work. The most proactive participation form, i.e., *initiating an innovation*, occurred most often in the human resources realm, e.g., in the cases HR-09 (introduction of measures to improve the work-life balance) and HR-04 (introduction of a new pension).

What was also predominantly found in these innovations was the works council’s *continuous participation* and contribution during the entire project, as the case of the introduction of performance reviews (HR-01) shows. Here, despite not being the initiator of the innovation, the works council contributed significantly to all relevant aspects: the official guideline for the superiors to conduct performance reviews was developed jointly by the works council and management, with the works council focusing on the scope of topics to be addressed during the review, the definition and phrasing of performance objectives as well as formalities (e.g., to whom the review sheets were to be sent after completion). Furthermore, the works council made sure that the superiors received training on how to conduct the reviews and instigated the option for employees to add a written comment to the review if they wanted to state their own perspective on certain points. Also, following the works council’s suggestion, an additional guideline was developed for the employees to help them to prepare for the review. Finally, the works council pursued integration of two more aspects: the employees’ option to negotiate their salary and to give feedback on their superiors’ leadership skills. These ideas were not implemented, however.

The human resources cluster of innovations also contains several cases that can be assigned to the third subdivision of content contributions, i.e., cases in which the works council came up with suggestions only for *specific aspects of the innovation*, but without being involved in the whole process. An example of this category is provided by case HR-06, the introduction of management by objectives: based on the works council’s proposal, the number of objectives per person was limited to three; additionally, the works council influenced the way specific goals were operationalized as well as the details concerning the variable salary component.

Contrary to the notable extent of content contributions in the human resources cases, *employee interests* were rarely explicitly addressed by works councils, probably because it seemed obvious which were at stake. This is mirrored in the finding that employees did not participate very much in these cases (Shajek, 2013, p. 156). An exception from the human resources participation pattern can be found in the two introductions of suggestion schemes (HR-11 and HR-12). Here, hardly any content contributions of the works councils could be observed. Instead, they were just accepted and supported, apparently as useful for all. We were hesitant to group these two cases into the human resources cluster; this result speaks for bringing suggestion schemes into a separate innovation cluster which may be called ‘establishment of innovation tools.’

#### Introduction of New Software

Software introductions (indexed IT-01 to IT-05) showed a contrasting action profile of works councils compared to human resources innovations. *Content contributions* were of minor importance whereas the bulk of participation could be assigned to the *employee-related activity dimension*. Within this dimension the category of *defending employees’ interests* was dominating: in all cases except for one (IT-01), company agreements were negotiated which interdicted performance and behavior monitoring through the new software. This has been a crucial and much-discussed demand of unions for decades (Klotz, 1987) and our cases seem to show that works councils are very familiar with the topic. Works councils were also concerned with protective regulations for overtime work caused through the software introduction (IT-01, IT-03, and IT-05). The *qualification of employees* category was represented, too: in two cases (IT-01 and IT-03), additional in-company training was negotiated.

The typical features of works council participation in the software cases are illustrated in case IT-03 (introduction of a new billing and customer data management system): though several works council members were part of the project team, they did not contribute novel ideas to the innovation. However, they raised awareness for several problems, e.g., that the new software system required more personnel, that the employees were concerned about the project, and that the staff of the consultant firm seemed to be incompetent. This kind of input corresponds to the *devil’s advocate* category, which represents a rather weak type of content contribution. By contrast, co-determination manifested itself strongly in the *employee-related* field. Several categories of this dimension were identified, with the defending of employees’ interests subdivision being the most prominent: the works council adapted an existing company agreement to ban the monitoring of employees’ work performance via the new software and made sure that overtime regulations were adjusted during implementation (i.e., employees could work overtime without a limit and recorded overtime did not expire). Furthermore, the works council initiated follow-up training for the staff, thus displaying activity in the qualification of employee category. Lastly, the works council discussed with the employees their concerns and complaints regarding the new software, so we also assigned the *communication with employees* category to this case.

#### Innovations Through Structural Changes of the Organization

Innovations that aimed at structural changes of the organization (indexed SC-01 to SC-08) showed another different action profile of works councils. As in software introductions, *defending employee interests* was at the forefront whereas *content contributions* were rare and concerned only few and very specific aspects (e.g., SC-02). A good example of this participation profile is provided by SC-02 (merging of several site kitchens into one centralized kitchen) where the works council concluded collective agreements with management to prevent layoffs and to set up new protective regulations regarding weekend work, shift work, and overtime. At the same time, only one idea came from the works council related to the content of the project: providing the employees with new work clothes, e.g., thermal jackets and shoes for the deep-freeze stores. Yet, in contrast to software introductions, works councils contributed to *enhancing the context parameters* in some cases, e.g., SC-07 (launching the production of a new product at the plant): here, the works council acquired public funding for the expansion of the plant’s infrastructure and staff retraining. In addition, the works council advertised the project to management and public authorities to receive support for the plan.

What also occurred more often in the structural change projects than in other case types was that the works council actively supported and promoted the innovation vis-à-vis the employees as SC-08 (restructuring of the corporate customer department) or SC-04 (converting a customer service call center into a separate service company) showed. Employees did not participate very much in these cases because, here, the most negative consequences, like workplace losses, could occur which precludes discussion as a possibility (Shajek, 2013, p. 155).

#### Workflow Innovations

Innovations dealing with workflow changes of the organization (indexed WF-01 to WF-08) showed yet another action profile of works councils: *content contributions*, *protection of threatened employee interests*, as well as active support and *promotion of the innovation* were equally found. In WF-06 (implementation of an efficiency enhancement program) all these fields of participation were on display. A member of the steering committee as well as of all subprojects, the works council was continually and proactively involved in the relevant content-related topics of the innovation, especially in improving the efficiency of various divisions like sales, services, technology, infrastructure, etc. Due to the goal of the innovation, it became apparent soon that jobs would be at stake. The works council, however, averted immediate layoffs in turn for accepting staff reductions through a social compensation plan and attained the preservation of jobs for disabled persons. Despite inevitable job losses, the works council still campaigned for stronger acceptance of the project because its overall advantages seemed to outweigh the disadvantages. Workflow changes may offer a larger scope for the works council than structural changes, which might explain this participation pattern. In none of the 8 cases, an additional training for employees was required. There might have been no need for a special training. In the workflow innovations, employees participated most strongly and in manifold ways because their special know-how was needed and their personal interests were at stake (Shajek, 2013, pp. 155, 156).

Of course, the influence of the innovation type on the actions of works councils is limited. Looking into the details, all cases differ at least somewhat from all other cases. The characteristics of the firm, of the leading managers, works councilors, and of the innovation itself make each case unique. Even very similar projects within the same firm can be handled quite differently by the works council. For instance, the three projects of improving the quality and efficiency of a hospital’s services (WF-01 to WF-03) are handled quite differently by the works council: The works council initiates WF-01 and is involved as a member of the project team. This project ends with a positive social success and a negative economic success. In WF-02, the works council is also directly involved in a kind of co-management; here, the project ends with a negative social success and a positive economic success. In WF-03, the works council is “not very present” in the project while intensive employee participation is organized by the management. This project is judged neutral regarding its social success and very positive in its economic success. Comparing only these three adjacent projects, the different success rates cannot be consistently related to the actions of the works council or the management and are probably more dependent on the specific problems surrounding each project.

Table 2 gives a condensed overview of the qualitative results of works council participation.

#### Works Councils’ Consultations With Employees and Resistance Against Innovations

Consultations between works council members and employees about the upcoming innovation were only reported in 11 cases, sometimes primarily with employees providing information for the works council and sometimes in the other direction. From the literature, one would have expected to find this kind of interaction and exchange in more cases. It seems that the works council stays constantly in touch with relevant employees such that a special consultation during the innovation process is often not necessary or worth reporting.

Even rarer are the 6 cases with acts of resistance and opposition from the works council. Opposition against the innovation or its parts (WF-05, WF-07, and HR-02) is sometimes combined with intensive content contributions (HR-06) and sometimes used as a negotiation tactic while pursuing other goals (SC-06 and IT-04).

The participation of the employees was small in many cases. Often, they were only informed about the innovation at work meetings, which were either held jointly by management and works council or separately, as reported in eight cases. At these meetings, they could ask questions and express their approval or disapproval. However, it is hard to assess how considerable the influence of these meetings was on the final result (Shajek, 2013, pp. 152–154).

#### Works Council Participation in Successful and Less Successful Innovations

A final interesting aspect is the relation between innovation success and the kind of works council activities. For the success assessment, the combined success questions of the questionnaires from both sides are used. Most of the innovations lie above zero, i.e., they were at least a bit successful. The upper quarter of the more successful 9 out of 35 innovation cases had a value close to one or larger (>0,90) on the scale from −3 to +3 (see SC-04, SC-06, SC-08, WF-04, WF-06, HR-02, HR-08, HR-09, and HR-14). The 8 least successful cases, the lowest quarter, had values <0 (SC-02, SC-03, SC-05, WF-01, WF-05, HR-11, HR-12, IT-03, and IT-05). It may be noted that none of the software introductions was fully successful; on the other hand, all human resources innovations are at least partly successful (if the two idea management introductions, HR-11 and HR-12, are taken out of the human resources category, see above). Additionally, from the above reported 11 cases of works council/employee consultations, 5 belong in the successful category, 6 in the partly successful group, and none in the less successful group.

The comparison delivers an interesting picture: the successful innovations often had strong content contributions and strong innovation support from the works council, and were not centered around the protection of employees’ interests. The works councils involved in the unsuccessful innovations brought almost no content contributions, did not support these innovations, and concentrated their work on threatened employee interests. The differences of co-determination in successful vs. less successful cases are illustrated by two examples. HR-14 was a successful innovation with the aim to introduce a trust-based working time regulation, i.e., flextime without any recording of working hours. The idea for the project was initiated jointly by the works council and management. The works council was also continually involved in the development and implementation of the innovation: In a first step, the works council and the project leader defined the rules of the new trust-based working time regulation, e.g., that working hours would not be recorded or that team members would have the obligation to coordinate their working time with each other to get tasks done. In the following step, the works council and project leader launched and evaluated three pilot projects in different divisions of the company to test the concept. While the feedback from the participants of the pilot projects was positive, concerns among both employees and superiors were growing. Employees feared that future overtime work would be concealed by the new regulation and superiors suspected that employees would use it to work less. The works council, however, was in constant talks with both groups throughout the project, thus being able to dispel their concerns and win them over.

By contrast, in WF-05, a less successful project on deregulating and streamlining work processes, the works council was almost exclusively dealing with defending employees’ interests. He made sure that the job-related activities were not evaluated on the individual employee level, but only on the team or department level so that tracing of individual performance was avoided. The works council also limited the number of performance indicators that would be recorded. Since as a consequence of the project, staff reductions were anticipated, the works council tried to avert layoffs (partly achieved) and even showed acts of resistance against the innovation by recommending employees to fill in activity lists inaccurately.

Of course, this picture is based on a relatively small sample size; no causal conclusion can be drawn from this comparison. Yet, with some caution, a causal test can be tried with the quantitative data and a path analysis.

### Quantitative Analysis

First, descriptive summary data of the model variables are presented in Table 3.

**TABLE 3:** Descriptive data of the model variables.

Variable	*M*	*SD*	WCI	WCP	EP	KG	CC	IS
Works council influence (WCI)	4.27	1.01	1					
Works council participation (WCP)	3.40	0.99	0.27^†^	1				
Employee participation (EP)	2.71	0.76	0.21	0.47**	1			
Knowledge growth (KG)	3.73	0.71	–0.05	0.02	0.29^†^	1		
Coordination capability (CC)	4.04	0.81	0.09	0.26^†^	–0.09	–0.09	1	
Innovation success (IS)	0.55	0.58	0.08	0.39**	0.30*	0.40**	0.48**	1

**p < 0.01, *p < 0.05, ^†^p < 0.10; n = 44.

The mean values for top management and works council influence on the scale from 0 to 6 are *M* = 5.39 and *M* = 3.66, i.e., works council influence is on average 1.73 units lower than management influence. Transposing this negative difference into a positive influence value by adding the scale range of 6 units gives a purely technical average works council influence value of *M* = 4.27 (see Table 3). The average intensity of works council participation during the innovation is *M* = 3.40, i.e., above the scale mean of 3, whereas the average score of *M* = 2.71 for employee participation lies below that scale mean. Knowledge growth and coordination capability are assessed quite high with *M* = 3.73 and *M* = 4.04, respectively. Innovation success, which was measured on a scale from −3 to +3, averaged just above the scale mean of 0. The standard deviations vary between 3/4 and 1 scale units, and only innovation success has a low standard deviation of *SD* = 0.55, i.e., half of the innovations lie between 0 and 1. Since success estimations are often a bit upward biased (Scholl, 2004, pp. 15–20, for a validation method mix), about half of the researched innovations are just partly successful, whereas the more successful and the less successful ones each represent one-quarter of the sample (see the preceding paragraph). Thus, this sample mirrors our intention to get a broader array of innovation success for a better examination of relevant success factors.

**FIGURE 2:**
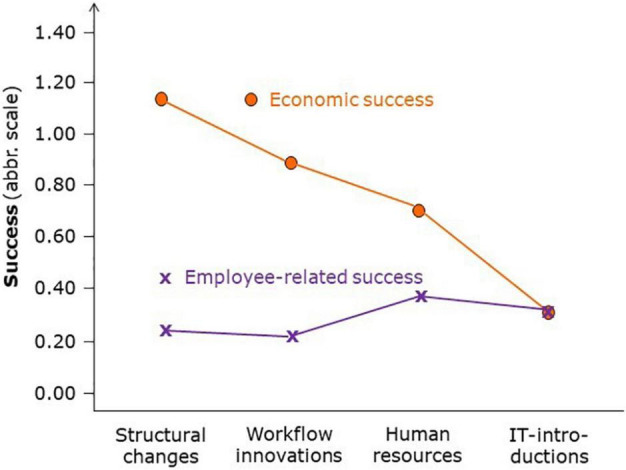
Average economic and employee-related success in the four innovation types.

A more detailed picture of innovation success in the different innovation types on the one hand and the beneficiary on the other is given in Figure 2.

Economic success is estimated higher than employee-related success in three of the four types; only IT innovations showed both beneficiaries with the same (low) success. And whereas there are clear economic success differences for the four types with structural changes as most successful, employee-related success was similar in all four types – and not very impressive. Table 4 shows, even more convincingly, that economic and employee-related success often differs within the single case. While 13 innovations ended with similar results for economic and employee-related outcomes (3 losses, 8 partly successful, and 2 fully successful), the other 22 cases brought about different economic and employee-related success with 15 favoring economic success and 7 favoring employee success.

**TABLE 4:** Distribution of economic success and employee-related success.

Employee success					Σ

	Economic success	<0	0–0,9	>0,9	
<0		3	2	4	9
0–1		5	8	9	22
>1		0	2	2	4
	Σ	8	12	15	35

*The categorization is the same as in the combined estimates.*

Whereas 15 cases were economically quite successful (*M* > 0,9), the same holds only in four cases for employee-related success. Within the overall success cases, only 2 have values of *M* > 0,9 for economic and employee-related success together. Likewise, among the overall less successful cases, there are only three where both subcategories have values of *M* < 0.

**FIGURE 3:**
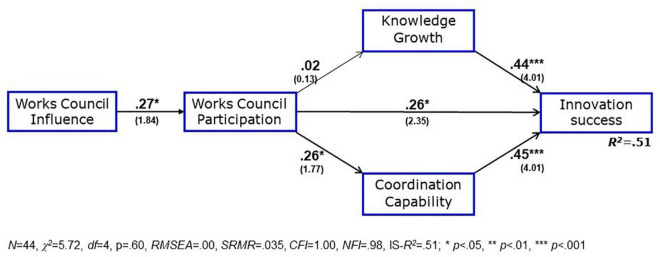
Test of the theoretical model from Figure 1. Numbers in bold face next to paths are path coefficients, *t*-values are given in brackets below; *R*^2^ = explained variance.

Table 3 also exhibits the correlations between the model variables. They show medium to high values with innovation success, except the distal variable of the overall influence of the works council. This is a happy omen for the path analysis which is shown in Figure 3.

As can be seen from the indices below Figure 3, the empirical data are almost perfectly in line with the theoretical model from Figure 1: The χ^2^-value with *p* = 0.60 shows that the empirical estimate does not significantly deviate from the theoretical assumptions of the model; the critical *RMSEA* index is zero, and the comparative fitness index (*CFI*) reaches the maximal value of *CFI* = 1.00, while the less biased normed fitness index (*NFI*) is almost as high with *NFI* = *0.98*. Yet the β coefficient of the path from works council participation to knowledge growth is near zero and not significant, and H2 is not confirmed. Skipping this path cannot improve the already perfect alignment of the empirical data to the theoretical model.

**FIGURE 4:**
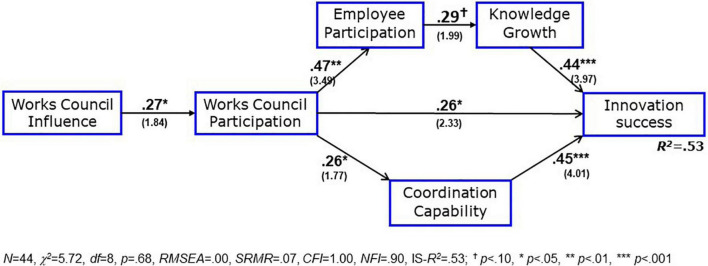
Enlarged model of participation and innovation success. Note see Figure 2.

Now, employee participation is introduced into the model, directed by an inspection of the correlation matrix. Employee participation shows a high correlation with works council participation (*r* = 0.47, *p* < 0.01) and – different from the works council participation – a weakly significant correlation with knowledge growth (*r* = 0.29, *p* < 0.10 versus *r* = 0.02). With these hints, we can specify the expectation E1 that the amount of employee participation has a positive effect on the innovation process: We test this expectation by using employee participation as a mediator between works council participation and knowledge growth, see Figure 4.

The fitness indices of this revised model are very similar to the former, only the *NFI* is somewhat lower. On the other hand, degrees of freedom are twice as high (*df* = 8 instead of *df* = 4); that gives a stronger model test which deviates even somewhat less from the enlarged theoretical model (*p* = 0.68 instead of *p* = 0.60). Works council participation has now an indirect total effect of *β* = 0.14 (*p* < 0.10) on knowledge growth via employee participation, which replaces the refuted H2. The total effect of employee participation on innovation success is *β* = 0.13 (*p* < 0.10). [An additional positive effect of employee participation on the innovation success via less resistance of *β* = 0.12 (*p* not reported) was found by Shajek, 2013, p. 177]. Confirming the hypotheses H1 and H3, works council participation has positive effects of *β* = 0.26 (*p* < 0.05) on innovation success as well as *β* = 0.26 (*p* < 0.05) on coordination capability; the latter mediates a combined effect of *β* = −0.35 × −0.35 = 0.12 on innovation success. The total effect of works council participation on the success of innovations, including the indirect effects, via knowledge growth and coordination capability is with *β* = 0.44 (*p* < 0.01) as positive as that of the direct determinants, knowledge growth, and coordination capability. Together, the three direct paths raise the explained variance of innovation success to 53% (*R*^2^ = 0.53). This is an enormous effect, because a complete explanation could only utilize 80–90% of the variance, the rest are unavoidable measurement errors. The works council influence as a predictor for works council participation during the innovation (H6) can be confirmed, too (*β* = 0.27, *p* < 0.05). The total effect of the works council influence on innovation success is only indirect and much lower, with β = 0.12 compared to the total effect of works council participation with β = 0.44 (*p* < 0.01). This result supports the distinction of works council influence as a distal factor on the one hand and works council activities as a proximate factor on the other. Thus, as expected, the proximate factor is a much stronger predictor for innovation success.

Two further aspects have to be mentioned. First, the reported results are not the same for all four innovation types. Controlling the innovation type by means of a so-called cluster analysis (Muthén and Muthén, 2010, pp. 500, 501) may change the standard errors as basis for the significance tests, but cannot affect the path coefficients themselves. The cluster analysis shows a small deviation from the models in Figures 3, 4: The significance of the direct effect of works council participation on innovation success is reduced from *p* = 0.01 to *p* = 0.07, i.e., works council participation is more important for some innovation types and less important for others. A likely explanation for this innovation type dependence is the different extent of works council participation: structural changes and human resources innovations show a stronger participation (*M* = 3.60 and *M* = 3.61) than software introductions and workflow innovations (*M* = 3.03 and *M* = 2.99), although these differences are not significant. Knowledge growth and coordination capability are not dependent on the innovation type, they are equally important for all four types.

Secondly, a differentiation of innovation success into economic and employee-related success reveals some special effects (see the relevant correlations in Table 5). The former is important primarily for the economic prosperity of the organization and the latter for the well-being of the employees. First of all, the participation of the works council in the innovation process is positively correlated with both success types, yet a bit more for the employee-related success as the primary focus of works council activities. Employee participation, on the other hand, is primarily affecting the employee-related success, apparently together with relevant knowledge growth, as it is highlighted in the change from Figure 3 to Figure 4. Coordination capability, in turn, is more important for the economic success, which depends heavily on works council participation, as Figure 4 reveals. The separate inspection of the two subtypes of innovation success complements and differentiates the revised model from Figure 4.

**TABLE 5:** Correlations with subtypes of innovation success and their determinants.

Model variable	Economic success	Employee-related success
Works council participation	0.27*	0.33*
Employee participation	0.14	0.33*
Knowledge growth	0.15	0.48**
Coordination capability	0.45**	0.24^†^

^†^p < 0.10, *p < 0.05, **p < 0.01, one-sided, n = 44.

## Discussion

Before discussing the main results, it must be remembered that the study is about process innovations, not about the more often discussed product or service innovations. While works councils sometimes become active in these other innovations (e.g., Schwarz-Kocher et al., 2011), process innovations often demand more active engagement from works councils. In the following, we separately discuss the qualitative and the quantitative parts of the study but do, sometimes, include results gained from the other method.

### Qualitative Part

The qualitative interviews and their preparation and integration to 36 cases of works council participation in innovations exhibit a diverse landscape of co-determined actions. The innovation cases show the relationship between the works council and management on the operational level. They reveal that the legal rights provided for works councils by the German Works Constitution Act are in fact a poor predictor of what is actually happening during innovation processes; they just offer a frame and focus for action. Sometimes, the scope of works council participation goes beyond those rights, e.g., seeking support in the public sphere (SC-07), and sometimes stays behind, e.g., without contributions of own ideas (SC-01). Innovations involve many operational and managerial aspects, a thorough distinction of actions by referring to legal rights would be difficult and hamper the innovation process.

The innovation type has a marked influence on the kind of activities of the works councils. For each type, a different activity profile could be secured which answers research questions 1 and 2 from the literature overview. Works councils actively engage themselves with content contributions if chances are high that they can directly further employee interests, like in the human resources area, and less so, in the workflow cases. And they try to avoid possible violations of employee interests, especially in IT introductions, but also in structural and workflow changes. The IT introductions show that works council activities are also influenced by union guidance in that any work monitoring is excluded by contract (e.g., Klotz, 1987; Ziegler and Grobe, 2014).

Works councils are often less involved in detailed discussions (which is more typical for employee participation), but instead, they watchfully monitor the whole innovation process. They sometimes ask management for more and earlier information to employees (the lower forms of employee participation) and safeguard their direct objections against possible personal disadvantages (Shajek, 2013, p. 153). Like managers, in most cases, works councilors are examining whether the innovation unfolds in the preferred direction instead of participating in the daily task of the project members. It is noteworthy, especially for the European discussion, that the preferred direction of most works councils includes not only direct employee aspects, i.e., aiming at social success, but also the consequences for the firm, aiming at economic success. They may even defend staff reductions if it seems to be economically necessary, and only try to ameliorate the original management plan (e.g., WF-06).

Most cases show a lot of cooperation, whereas active resistance was rare and total opposition was not found at all. Works councils primarily try to safeguard employee interests, including those which are heavily dependent on the economic success of their firm like the number of workplaces. As a consequence, the works councils even promote innovations which lead to some losses for employees to secure the economic aim of the innovation (e.g., SC-04). On the other hand, employee-related losses (9) were not more frequent than economic losses (8, cf. Table 4). Works councils also contribute their own ideas, initiate sometimes innovations, and even actively promote some innovations vis-à-vis employees and other stakeholders. Such an active pro-innovation stance was found most often among successful innovations and was almost completely absent among the less successful ones.

Of course, these findings do not allow causal conclusions. Are innovations more successful if works councils participate more actively, or do they engage themselves only if they expect success? The bulk of results refutes the arguments of new institutional economics advocates (see the theory section of this article), who argue that only a pure shareholder orientation of management will secure long-lasting economic success, including innovation success; co-determination will at best not disturb such a policy, but is just too expensive. These arguments have already been refuted in other investigations (e.g., Scholl and Kirsch, 1986). Instead, in line with participation theory, our research confirms that works councils are not inhibitors but promoters of innovations in many cases, because innovations can secure the future competitiveness of their firm and its workplaces. Painstakingly observing employee interests and finding ways to align them with economic challenges is a win–win situation for firms and their employees, which answers research question 3.

The investigated cases deliver rich illustrative material for researchers and practitioners which could not be analyzed here at length. All cases are presented in the Appendix and give hints for the further improvement of industrial relations in the context of innovation processes. They also suggest that successful participation of works councils in innovations depends partly on formal and informal regulations, professional and personal competencies, and attitudes and proclivities; such detailed aspects demand further research. For instance, the interest in and competence of the establishment of innovation tools like idea management systems (HR-11 and HR-12) could be enhanced with a so-called ‘innovation promoter training’ (Hüttner and Scholl, 2011; Bierbichler and Scholl, 2014). The largest German union, IG Metall, invests heavily in its innovation competence (Ziegler and Grobe, 2014) and has successfully taken up this idea of an ‘innovation promoter training’ (Erb, 2016).

### Quantitative Part

While the qualitative analysis shows an abundance of facts and actions which can only roughly be categorized and summarized, the quantitative analysis concentrates *a priori* on selected variables, which are especially important and theoretically meaningful within a nomological network. For instance, the many different works council actions are condensed into one variable, the extent and intensity of works council participation. The theoretical model (see Figure 1) elaborates the causes and consequences of this key variable with one predictor and three consequences leading directly or indirectly to the target variable innovation success. Based on the combined assessments of management and works council members, this theoretical model was statistically almost perfectly confirmed; only the assumed direct contribution of works councils to knowledge growth for the innovation (H2) could not stand the test (see Figures 3, 4). Instead, an indirect effect via employee participation replaces this hypothesis. As a consequence, the above-mentioned role of the works council as a collective voice of the workforce (Freeman and Medoff, 1984; Freeman and Lazear, 1995) has to be modified: it is apparently not a messenger role for delivering critical information and alternative ideas of employees to management because they fear being sanctioned. It seems that the risk of employee voice is directly reduced through the visibility of their works council’s actions, such that employees become more courageous themselves; this may be especially important at work meetings where employee criticism, resistance, and opposition can be directly seen and heard by management and supervisors. The expectation E1, that there is a positive effect of employee participation on the innovation process, is confirmed by employee contributions to knowledge growth. Yet this holds especially for employee-related success as the correlations with employee participation and knowledge growth in Table 5 reveal. Direct employee participation is a worthwhile addition to works council participation, especially for themselves, because they know best what is needed for a good work life. That works councils enhance the knowledge growth for innovations (H2) can be accepted in the modified form of an indirect effect: works council participation safeguards employee participation, which enriches the knowledge base of innovations, especially that of software introductions and workflow changes, as was shown in the qualitative analysis.

The confirmed modified model from Figure 4 shows three significant influences on innovation success: Knowledge growth and a coordination capability are especially important for innovation success, as the path coefficients of β = 0.44 (*p* < 0.001) and β = 0.45 (*p* < 0.001) show, confirming H4 and H5. Both are positively influenced by an intensive participation of the works council, where knowledge growth is influenced indirectly (H2 modified, as discussed above) and coordination capability directly (H3). Coordination capability is especially important for the economic success of process innovations, a contrast to the primary importance of knowledge growth for employee-related success (see Table 5). Works council participation influences innovation success also directly with β = 0.26 (*p* < 0.05), confirming H1. An important part of this direct influence is probably the active support of innovations, which became manifest in the case material. If the indirect influences on innovation success via coordination capability and knowledge growth are added, the combined total impact of β = 0.44 shows a much stronger confirmation of H1. There are no other investigations into the unfolding dynamics of works council activities with which we can compare these results. But the strong effects of knowledge growth and coordination capability on innovation success found in the path model is absolutely in line with their effects in other innovation studies (So, 2010; Bentz, 2011; Scholl, 2014, 2019).

To judge the positive influence of works council participation in a broader context, three aspects are worth mentioning: (1) While works council participation, coordination capability, and knowledge growth are not the only determinants of innovation success, they do explain 53% of the larger part of the explainable variance (80–90%). (2) Looking at the explained variance of 53%, coordination capability and knowledge growth are not only enhanced by works councils but certainly also by management and especially by the innovation project members. Their combined influence can be estimated in the following way: the quantitative total impact of β = 0.44 by works council participation gives (squared) an explained variance of *R*^2^ = 0.19 (or 19%), i.e., about two thirds of the innovation success explanation (53–19% = 34%; 34%/53% = 0.64) come – via coordination capability and knowledge growth – from such other sources. (3) Our sample is probably a positive selection from the range of industrial relationships in larger firms. A more representative sample might give even larger effects than the β = 0.44. Less involved works councils would contribute less to the innovation success and employees might be more reluctant to contribute their ideas, making the strength of combined participation even look brighter. On the other hand, management might find other ways to stimulate a positive innovation climate while ignoring or corrupting a weak works council.

Comparing the results of the qualitative and the quantitative study, their special advantages and disadvantages compensate each other. The qualitative case studies deliver rich material about works council activities under the special conditions of the innovation type but hint on many other specific aspects which are too different to categorize and summarize. The introduction of questionnaire-based success estimates was a first step to show that works council participation is by and large positive for process innovations. The success measures could also illuminate the relation of economic and employee-related success for the four innovation types. Viewed from a European legislation angle, economic success is usually higher than social success even under relatively strong co-determination rights in Germany (cf. Figure 2). Yet the mainly cooperative stance of the works councils is also helpful for the represented workforce in that employee losses are confined and positive developments prevail. So, the same conclusion can be drawn for the theoretical discussion between new institutional economics (NIE) and participation theory (PT) from the qualitative material: works council participation does not endanger economic success; on the contrary, it promotes economic success, especially via strengthening the coordination capability as an important prerequisite (cf. Figure 4 and Table 5). The background assumptions of NIE should be changed from aiming at shareholders to include the other stakeholders, especially the employees (Donaldson and Preston, 1995).

The quantitative study cuts through the diverse qualitative descriptions with a few well-proven scientific constructs in order to get the essence out of these manifold processes. Thus, the quantitative study was able to predict the effect of works council participation with a few mediating variables on the success of the innovations and to explain a large part of its variance. Of course, these abstract constructs give only a few hints about action possibilities for practitioners or research gaps for scientists, which is the strength of the qualitative analysis. Yet, one aspect is especially noteworthy from the quantitative study for future research: Why does works council participation have only an indirect influence via employee participation on knowledge growth? The qualitative study hints at a low involvement into detailed discussions and at increasable consultations with concerned employees. Future studies should explore this aspect of works council participation.

Finally, the quantitative study confirms the theoretical usefulness of the chosen scientific constructs and recommends their use in other organizational research, including participation and innovation studies. A basic requisite of collaborative work is to combine a productive division of labor with a well-functioning coordination of the divided work pieces. For all complex problems, division of labor means to collect diverse relevant knowledge and still to keep the number of participants as low as possible. The more important and momentous decisions are, the more knowledge parts have to be assembled and integrated for reaching the growth of knowledge needed. Like managers who assemble and integrate relevant knowledge for their decisions from their subordinates, so do works councils from selected employees with a special view on the interests of the employees and the economic feasibility of their proposals. Employee participation is helpful, especially for assembling information, but the integration for the whole workforce has to be done by employee representatives like the German works council. The task of integration entails the coordination of several people with their information, experience, and opinions, for managers and works councils alike. It follows that knowledge growth and coordination capability are basic requirements of any complex collaborative endeavor, of participation regulations, innovation attempts, or any other organizational task. And it follows, too, that the participants of such a collaborative endeavor should have the necessary knowledge (parts), motivation, and legitimacy for participation. German co-determination apparently fares quite well in this regard with the election of works councils.

### Limitations

Although the quantitative study delivers a clear picture confirming largely the theoretical model, there are several limitations. The most important may be the relatively low number of cases for the quantitative study while it is relatively large for analyzing the qualitative material in detail. With a larger number of cases, the causal model could be extended with one or more variables, e.g., employee resistance (Shajek, 2013, p. 177). This may not only explain more variance in innovation success but also extend the degrees of freedom and thus, enable a stronger path analytic test of the causal order. Yet, even the small model with six variables integrates more causally ordered variables than in most experimental designs of participation and tests a broader integrated nomological net. So, it would be useful to enlarge the number of cases, especially with the developed questionnaire. We would be glad to help other research on further cases with access to our data. A second limitation is the necessity of imputing the data of missing industrial relation partners from the material of other respondents. Here, too, an enlargement of the number of cases with questionnaires from both sides would be helpful. The effects might become stronger, if 44 or more cases were obtained without any missing partner because imputation somewhat lowers the correlations. A third limitation is the minimal number of respondents per case. At least two respondents on either side, i.e., four per case, would raise the reliability and validity of the qualitative and quantitative data. A fourth limitation lies in the interview guideline which was very descriptive so that it was difficult to compare qualitative and quantitative results. While it is a strength to avoid interview biases which might prefer theory-consistent results, it is a weakness if concretizations of theoretical variables are not sufficiently included in the guideline; the latter pertains primarily to concrete phenomena of knowledge growth and coordination capability. Scholl (2014) showed how it is possible to investigate knowledge growth with interview data in a comparative way over quite different innovation cases by focusing on typical knowledge failures, called information pathologies.

## Conclusion

The critical stance, or at least skepticism of some scientists, especially economists, and of quite a lot of managers and their representatives against strong legal co-determination rights and an intensive participation of works councils are not warranted, as demonstrated once again for innovation processes by our study. Strong works council participation has not only a positive impact on the employee-related success of process innovations but even more on their economic success. This was convincingly shown with the quantitative data and the path analysis, and it was already visible in the qualitative case material. Both analyses go beyond existing research: The qualitative study reveals not only different degrees of works council and employee participation in innovations but also specific profiles of works council activity depending on the type of innovation. The quantitative study confirms the assumptions of several distinct hypotheses and their combination into a causal model. Both analyses show that looking only at the (non)existence of a works council is not suited to understand the consequences of co-determination, the legal form of indirect participation in Germany. Instead, it is crucial to take into consideration the kind and intensity of works council participation, its combination with employee participation, and the kind of innovation or – in general – the problems to be solved. This raises the question whether the concrete actions of employee representatives in other European and Non-European countries are perhaps more similar to the German case than the legal background. On the other hand, a different national culture may preclude similar actions and similar results. Co-determination is an enduring historical learning process (Welchowski, 1981). Multinational analyses can shed more light on these questions.

Judged from a practical point of view, the innovation cases show that works councils command a broad influence repertoire, from safeguarding threatened employee interests up to proactively co-managing innovations and thereby ensuring the future of the firm. It also became clear that they could profit from specific professional training regarding process innovations, and from more support of and exchange with employee participation and union expertise.

## Data Availability Statement

The raw data supporting the conclusions of this article will be made available by the authors, without undue reservation, to any qualified researcher.

## Ethics Statement

Ethical review and approval was not required for the study on human participants in accordance with the local legislation and institutional requirements. The patients/participants provided their written informed consent to participate in this study.

## Author Contributions

Both authors listed have contributed equally to the work and approved it for publication.

## Conflict of Interest

The authors declare that the research was conducted in the absence of any commercial or financial relationships that could be construed as a potential conflict of interest.

## Publisher’s Note

All claims expressed in this article are solely those of the authors and do not necessarily represent those of their affiliated organizations, or those of the publisher, the editors and the reviewers. Any product that may be evaluated in this article, or claim that may be made by its manufacturer, is not guaranteed or endorsed by the publisher.
