# Supplementary material for: Success of Process Innovations Through Active Works Council Participation
Source: Front Psychol. 2022 Apr 8;13:795143. doi: 10.3389/fpsyg.2022.795143 (PMC9024309; doi:10.3389/fpsyg.2022.795143)
Supplement: Supplementary file 1 [file Data_Sheet_1.docx]

| **,,Short characterization of the innovation cases  from management and works council interviews**  **Works Council Participation / Employee Participation^[[1]](#footnote-1)^**  **Cases^[[2]](#footnote-2)^** Abbreviations: D = Directorate, MG = Management (representative),   WC = Works Council (representative), EM = Employees  **I. Innovations through structural changes of the organization** | | | |  | | | |
| --- | --- | --- | --- | --- | --- | --- | --- |
| Converting an internal training center for EM into a training academy that operates on the free market.  (SC-01)  0,41 / 0,10 / 0,75 | - WC keeps a watchful eye on the project, but without contributing additional ideas to the concept - No WC involvement in the project group - Approval of decisions in the superordinate steering committee - / Shortly before implementation EM are informed about the project by MG upon WC’s demand, - / Individual EM are requested to provide knowledge and information that is required for the project | | | |  |  |  |
| Merging several site kitchens into one centralized kitchen  (SC-02)  -0,20 / -1,20 / 0,80 | - / EM are informed by WC-bulletin about the upcoming project - A new collective agreement is concluded between WC and MG (no lay-offs in return for reduced Christmas and vacation pay) - WC initiates new regulations on work clothing (thermal jackets, shoes) - Further agreements are concluded between WC and MG on weekend work, shift work, overtime - / EM are informed by MG only one year after project start - / Roundtable discussions with EM (moderated by external consultant) - / Certain EM provide knowledge and information to the project group | | | |  |  |  |
| Merging several site laboratories into one centralized laboratory  (SC-03)  -0,35 / -0,20 / -0,50 | - Continuous proactive WC participation regarding the concept- and personnel-related topics of the project - WC identifies weak points of the project - Strong WC participation especially in personnel calculations - / EM are informed by MG about the project after concept is finalized - / WC collects information from some individual EM - / EM are kept updated about the project through WC and MG at regular staff meetings - / Selected physicians are involved in process planning | | | |  |  |  |
| Converting a customer service call center department into a separate service company  (SC-04)  1,08 / -0,20 / 2,35 | - WC is kept updated extensively about the project without contributing own ideas - / MG informs EM at regular staff meetings about the progress of the project - Negative consequences are openly discussed between WC and EM - WC consents to considerable salary and vacation losses - WC defends the project vis-à-vis EM, while widely ignoring EM concerns | | | |  |  |  |
| Takeover of a production site from a competitor  (SC-05)  -0,05 / -0,50 / 0,40 | - Project group with D, MG, WC / partly EM - / General information of EM by WC and MG via intranet - / An EM group assert claims vis-à-vis WC, goes public - WC negotiates and concludes transition agreement (EM benefits were either left unchanged by the new owner or compensated by additional payments) - WC gets MG to guarantee preservation of existing jobs for the next five years | | | |  |  |  |
| Integrating surplus personnel into the company by establishing an additional department  (SC-06)  1,35 / 1,4 / 1,3 | - / Project originates from a group of disgruntled employees - WC induces the affected EM to form a group of spokespersons - WC asks EM to contribute their own ideas and suggestions - WC averts salary losses - WC secures status of affected EM as part of the core workforce instead of dispensable surplus workforce - WC threatens MG to cause the failure of the project if certain demands are not met - WC critically questions the concept of the project - WC offers EM one-on-one counselling | | | |  |  |  |
| Launching the development and production of a new product at the plant (SC-07)  0,45 / 0,90 / 0,30 | - WC acquires public funding for staff retraining and expansion of the infrastructure - WC advertises the project to MG and state authorities - / EM are informed by WC about the project at a staff meeting, - Selected EM are asked to contribute information about technical details | | | |  |  |  |
| Restructuring of the corporate customer department  (SC-08)  1,50 / 0,70 / 2,30 | - Continuous proactive WC participation in all aspects of the project by WC members joining expert teams and project groups - WC initiates the implementation of short lines of communication between WC and MG during the project - Conflicts of interests between MG and EM are settled: WC actively promotes project vis-à-vis EM (WC hosts kick-off event together with MG, a WC member is in charge of project-related internal communication). | | | |  |  |  |
| **II. Workflow innovations** | |  | | | |  |  |
| Improving quality and efficiency of a hospital’s medical service  (WF-01)  -0,12 / 0,41 / -0,65 | - WC is member of the project team and thus involved in all related issues. - / EM are informed about the project at a works meeting a few weeks after project started. - WC attempts to convince the physicians to participate in the project in one-on-one meetings and asks them to provide accurate information about their job-related activities). - WC attempts to create transparency about the project in order to prevent the physicians from refusing to be evaluated. - However, many physicians still refuse to cooperate | | | |  |  |  |
| Improving quality and efficiency of a hospital’s nursing service  (WF-02)  0,60 / -0,60 / 1,80 | - / EM are informed prior to project start at a works meeting and via staff newspapers. - A WC member from the nursing staff participates continuously and proactively in all content-related topics as member of the project management team - MG and WC collaborate closely (co-management), such that the head of project management team is partly cut off from information - WC approaches and selects qualified EM for participation in project groups - / Company’s suggestion scheme is used to collect further ideas for the project from EM - WC gives impulses on the topic of "Healthy aging with a hospital job". - WC initiates a follow-up project on the reorganization of the hospital’s medical services (cf. WF-01) | | | |  |  |  |
| Improving quality and efficiency of a hospital’s physiotherapy unit (WF-03)  0,53 / 0,01 / 1,06 | - WC "not very present" during project, no contribution of own ideas - WC primarily functions as approval body for MG’s decisions - / EM are being informed at project start - / On MG’s initiative, all EM participate in the project extensively at quarterly meetings, roundtable discussions, and team sessions - / In task groups EM prepare proposals on how to improve efficiency of outpatient and inpatient routines - / roundtable discussions are held with all EM on the anticipated negative consequences of increased efficiency | | | |  |  |  |
| Reducing the makespan in production  (WF-04)  0,95 / 0,35 / 1,55 | - Strong WC participation in the "Change Management" subproject - / EM are being informed at the beginning of the project, further information is given via bulletin boards, intranet, e-mail, and at works meetings. - Due to EM persistent criticism, WC initiates a kick-off event for EM in the middle of the project - WC promotes the project vis-a-vis EM - WC facilitates introduction of shift work and sets up regulatory framework - WC raises discussions about possible disadvantages for EM ("worrywart role") - / Selected EM are involved in the project for analyzing and finding solutions for specific problems | | | |  |  |  |
| Deregulating and streamlining work processes  (WF-05)  -0,07 / 0,10 / -0,25 | - / At the beginning, the external consultant conducts numerous interviews with EM affected by the project. - / EM are asked to complete job-related activity descriptions - / EM are informed about the project by WC and MG only after the decision for the project has been made - WC makes sure that the job-related activies are not evaluated on the level of the individual EM, but only on the team or department level; thus, tracing of individual performance is avoided. - WC co-determines with MG which performance indicators should be recorded - WC recommends EM to fill out activity lists inaccurately => "opponent role" - WC advocates preservation of the existing jobs and proposes a job guarantee for two years. | | | |  |  |  |
| Cost savings through a corporate-wide efficiency program  (WF-06)  0,95 / 0,15 / 1,75 | - Continuous and proactive WC participation regarding the concept and all personnel-related topics of the project (WC members in all subprojects and in the steering committee). - / EM are already informed at the start of the project, further information followed via e-mails, newsletters, intranet and on works meetings. - WC in a "positive corrective role". - WC campaigns for stronger acceptance of the project (due to some departments’ initial refusal to participate in the analysis) - WC averts immediate lay-offs in turn for accepting staff reductions in socially acceptable way - WC attains preservation of jobs for disabled persons - / After final decision on the implementation of various measures, further meetings of MG and WC with EM are held | | | |  |  |  |
| Introduction of a change management project to support a corporate-wide efficiency program  (WF-07)  0,46 / 0,39 / 0,52 | - Project is initiated by WC, but implementation is made impossible due to a similar project pursued by MG - / EM are being informed in advance of the project by WC and MG in works meetings. - No further involvement of WC and EM | | | |  |  |  |
| Introduction of parking space management for employees and visitors (WF-08)  0,49 / -0.09 / 1,06 | - WC initially rejects the project and thus causes delay for approximately 2 years; after arbitration committee decides in favor of management, WC can’t avoid any longer to deal with the issue. - / EM are informed via WC bulletin after conception of the project is finalized. - WC attains exemption from parking fees for those employees who forgo salary as part of a reorganization program - Mutual agreement of WC and MG on personal data protection (video surveillance data of the parking barriers may not be used to monitor EM working hours or other work-related behavior). | | | |  |  |  |
| **III. Human resources innovations** | |  | | |  |  |  |
| Introduction of regular employee performance interviews  (HR-01)  0,62 / 0,45 / 0,81 | - WC primarily responsible for the content as well as formal aspects and organizational procedure of the employee performance interviews - / EM are being informed before project implementation at a works meeting - WC ensures that managers receive training on how to conduct the interviews - WC ensures that EM have the option to give feedback and comments on their performance evaluation that results from the interview - WC restricts managers’ possibility of expressing harsh criticism during the interviews | | | |  |  |  |
| Introduction of a new work time regulation  (HR-02)  1,25 / 0,40 / 2,10 | - WC rejects MG’s attempt of introducing trust-based work time (i.e. no recording of work time) because overwork of EM is anticipated - WC insists on an agreement with MG for transparent rules of recording and compensating overtime. - WC enforces separate agreements for dealing with overtime demanded by MG on the one side and overtime by EM choice on the other side. - / EM are informed about the project only by the time it is implemented. | | | |  |  |  |
| Harmonizing the wages of blue and white collar workers (HR-03)  0,10 / 0,80 / -0,60 | - / EM are informed by WC and MG after final decision for the project - WC interviews selected employees in the run-up to the project. - WC advises and motivates EM to file an objection against their new wage classification in order to achieve a higher classification - In a joint committee WC and MG negotiate about EM objections against their wage classification and more accurate or appropriate classifications. - / Comprehensive project communications concept with flyers, posters, and intranet; regular updates on interim results are provided. | | | |  |  |  |
| Improvement of company pension scheme (HR-04)  0,69 / 0,40 / 0,99 | - Initiative comes from the WC - WC achieves increase of step-up amount for beyond-tariff employees - WC demands that employer’s payments be raised for the pension fund - / EM are informedat a works meeting after final decision for the project and selection of the pension fund provider. - / EM make alternative suggestions for a pension scheme which are reviewed and finally rejected by MG | | | |  |  |  |
| Introduction of a periodical employee survey  (HR-05)  0,40 / 0,50 / 0,30 | - WC reviews the questionnaire with regard to content and participants’ anonymity - On WC’s initiative, questions regarding "corporate health management" and "work-life balance" are added to the survey - WC is involved in defining the circle of participants (including managers) - WC participates in deciding which measures are implemented as a result of the employee survey - / Employees are informed by a letter from D and later on by supervisors - WC calls on EM to participate in the survey. - / Selected EM participate in focus groups in order to discuss the survey results. | | | |  |  |  |
| Introduction of management by objectives  (HR-06)  0,43 / -0,30 / 1,20 | - WC openly objects against MbO (which is part of a collective labor agreement) because wage reductions through the back door are suspected - / EM are informed about the project by the time the collective agreement is published - WC informally asks EM about potential problems and risks of MbO prior to its implementation - WC calls on EM to oppose against the project - On WC’s initiative the number of objectives per person is limited to 3 - WC is involved in setting standards on how to define and operationalize the objectives - WC is involved in defining the variable component of the salary | | | |  |  |  |
| Prevention of psychological stress  (HR-07)  0,55 / 0,70 / 0,40 | - WC initiates project, but is hardly involved during implementation - / EM are informed by both MG and WC directly at the start of the project - / EM are kept updated by external project management during works meetings. - / Many interviews are conducted with EM about their needs. - A WC member is assigned to participate in a stress management training in order to check its quality. | | | |  |  |  |
| Improving the compatibility between working life and family life  (HR-08)  1,11 / 1,37 / 0,88 | - WC continuously and proactively participates by suggesting ideas on all aspects of the project. - / EM are being informed at the beginning of the project during works meetings - / EM receive further information via flyers, employee magazine, and by WC via intranet - / In several workshops EM are asked about their needs. - WC strongly advocates the expansion of qualification offers for EM during parental leave (e.g. trainings, regular employee interviews) - WC has considerable influence on the following topics: financial and material benefits for EM, new working time arrangements, re-employment guarantee for EM after parental leave regardless of length of company affiliation, certification for the "Work & Family" program | | | |  |  |  |
| Improving the work-life balance (HR-09)  1,60 / 1,40 / 1,80 | - Project is initiated, planned and implemented under WC’s almost sole responsibility - / After finalizing the concept, the project is presented to the EM at a works meeting by the WC and the HR department. - / EM survey on satisfaction with the project is conducted. | | | |  |  |  |
| Introduction of a critical incident reporting system  (HR-10)  0,85 / 0,40 / 1,30 | - WC is member of the project team and thus involved in all content-related questions - WC takes a critical perspective on the project ("typical WC") - After concept is finalized, WC informs EM about upcoming project at a works meeting - / Selected EM become members of the project group and help implement the reporting system | | | |  |  |  |
| Introduction of a new ideas management system (HR-11)  -0,20 / -0,40 / -0,00 | - WC contributes only few suggestions - / EM are informed by plant MG at kick-off event - WC initiates an "Innovation Day" to familiarize EM with the new ideas management system and to promote its use - / Further project-related WC communication towards EM via e-mails and special campaigns | | | |  |  |  |
| Introduction of an ideas competition  (HR-12)  -0,20 / 0,40 / -0,80 | - No significant contributions by WC - WC limits its involvement to complying with formalities by drafting a company agreement - / EM are informed via e-mail shortly before agreement becomes effective. - / Further information for EM at subsequent staff meetings | | | |  |  |  |
| Introduction of a sales bonus for sales employees  (HR-13)  *no data* | - WC demands from MG to pay every EM a guaranteed minimum bonus (eventually granted for the 1st year after introduction only) - WC and MG jointly specify the conditions under which the sales bonus is granted together - / WC and MG seek EM advice on the concept - WC and MG mutually agree on the provisional nature and modifiability of the bonus system in case of emerging problems - / By the time the sales bonus is implemented, EM are informed in a works meeting | | | |  |  |  |
| Introduction of trust-based working time (HR-14)  1,25 / 0,75 / 1,75 | - Idea jointly developed by WC and MG - WC involved entirely in the development and implementation processes - WC dispels EM fears that overtime will be concealed by trust-based working time - WC dispels MG concerns that EM will use trust-based working time to work less. | | | |  |  |  |
| Employee-friendly redesign of the formal warning process (HR-15)  0,80 / 1,00 / 0,60 | - WC initiates the project as a reaction to EM suggestions and complaints - Concept is developed and substantiated by WC alone - WC offers for open talks with MG are declined by the latter as no need for change is seen - Innovation eventually gets stuck and is never implemented | | | |  |  |  |
| **IV. Introduction of new software** | | |  | | | |  |
| Introduction of 3D design software  (IT‑01)  0,60 / 0,90 / 0,30 | - WC and MG conclude company agreement on compensation of overtime during software introduction - / EM are informed about the introduction by WC and MG at works meetings.  They are asked to support the project team. - WC attains a four weeks software training for all EM (MG suggested 2-week  training for only some of the EM) - / Certain EM are selected by MG as key users who are trained more intensively and act as multipliers - WC and EM jointly develop a concept for organizing the work steps and processes with the new software - / WC asks ca. 10% of EM to give a critical evaluation - WC encourages EM to work with the new software | | | |  |  |  |
| Introduction of software for more efficient and standardized teamwork  (IT‑02)  0,57 / 0,51 / 0,61 | - WC participates in project meetings, but is not member of the project team - / The negotiating committee of WC and MG calls in some EM for further advice - Mutual agreement of WC and MG to ban performance and behavioral monitoring by means of the new software - / EM are informed via intranet and e-mail about WC-MG negotiations prior to the introduction of the software; later on, more information is provided at subsequent works meetings - WC enforces a mechanism to protect users from work overload (orders placed via the system must be confirmed by telephone by the contractor) - / A group of dedicated EM collect information on their own initiative, prepare  user information, and give small presentations | | | |  |  |  |
| Introduction of a new billing and customer data management system (IT‑03)  -0,55 / -0,25 / -0,85 | - Some WC members are members of the project team and in this role they contribute only little content input, but indicate implementation problems - / EM are informed at works meeting by MG prior to the implementation of project - / EM are also informed about the status of the project via e-mail and the employee magazine - WC uses an existing company agreement to ban monitoring of EM performance and other work-related behavioral controls via the new software - WC secures that overtime regulations are adjusted during implementation (i.e. EM can work overtime without a limit and recorded overtime does not expire) - WC initiates a key user system and follow-up trainings for EM - / Ca. 25% of EM become test users, some EM are selected for project team - / Skilled and experienced users are consulted by the project team about technical issues on a case-by-case basis | | | |  |  |  |
| Introduction of a billing system  (IT‑04)  0,80 / 0,13 / 1,51 | - Kick-off with WC, but without EM - WC regulary attends meetings of the project management team, mainly for informational reasons, less for contributing own ideas - WC initiates a company agreement to ban performance monitoring with the new software - WC threatens MG to deny approval of the new system - / EM are informed about the project after the concept is finalized - / Further information for EM is provided via intranet, company newspaper, MG newsletter, by MG directly, and at works meetings - / Selected EM are deployed as key users and test users at the beginning, later as dissemnators - / MG initiates training for all EM - / EM feedback is documented on the intranet | | | |  |  |  |
| Introduction of new software for machine control (IT‑05)  0,40 / 0,50 / 0,30 | - WC is continuously and proactively involved in technology-related and personnel-related project topics => double function as WC and as project member - / EM are initially informed by MG at a works meeting. - / After consuling with WC, MG provides further information for EM via intranet, newsletter, company magazine, and works meetings held by the project manager - WC and MG conclude agreement on the limitation and appropriate remuneration of overtime during the software introduction - Additonal agreement to ban performance and behavioral monitoring through the new software - / In the design phase, the EM at foreman level support the project team - / Further training is offered for all affected EM and additional training for key users - / EM openly reject motivational training provided by an external consultant; another motivational event is offered by MG | | | |  |  |  |

1. Descriptions about employee participation are taken in reduced form from Shajek (2013, pp. 139-147) as supplements; they are introduced with a slash: / [↑](#footnote-ref-1)
2. Case numbers in brackets are often referred to in the text. The success measures, scaled from -3=total failure up to +3=total success, are given below: Overall Success = first number, Employee related Success = second number, Economic Success = third number. [↑](#footnote-ref-2)
